# Risk factors for diaphragmatic dysfunction in mechanically ventilated patients: a systematic review and meta-analysis

**DOI:** 10.3389/fmed.2026.1828707

**Published:** 2026-07-30

**Authors:** Ruixin Che, Shiqin Pan, Jinhai Han, Qin Ma, Ailing Xue

**Affiliations:** 1 Qinghai University, Xining, China; 2 Department of Critical Care Medicine, Qinghai Provincial People's Hospital, Xining, China

**Keywords:** diaphragmatic dysfunction, mechanical ventilation, meta-analysis, risk factors, systematic review

## Abstract

**Objective:**

To systematically identify and evaluate the risk factors for diaphragmatic dysfunction (DD) in mechanically ventilated patients.

**Methods:**

A comprehensive search of PubMed, Embase, Web of Science, CNKI, Wanfang, VIP, and CBM was conducted from database inception through July 2025. Case–control, cohort, and cross-sectional studies reporting risk factors for DD in mechanically ventilated patients were eligible. Two reviewers independently screened records, extracted data, and assessed risk of bias. Meta-analyses were performed using Review Manager 5.4.

**Results:**

Eleven studies comprising 5,272 mechanically ventilated patients were included (7 cohort, 1 case–control, 3 cross-sectional). Meta-analysed risk factors: infection/sepsis (OR = 2.65, 95% CI: 1.32–5.34, *p* = 0.006), higher APACHE II score (OR = 1.19, 95% CI: 1.09–1.30, *p* < 0.0001), older age (OR = 1.02, 95% CI: 1.00–1.04, *p* = 0.02), and higher BMI (OR = 1.03, 95% CI: 1.01–1.06, p = 0.02) were each independently associated with increased DD risk. Meta-analysed protective factors: higher serum albumin (OR = 0.88, 95% CI: 0.79–0.98, *p* = 0.018) and greater grip strength (OR = 0.70, 95% CI: 0.62–0.78, *p* < 0.0001) were protective. Narrative-only findings: MV-related exposures could not be pooled owing to incompatible operationalisations across five studies; surgery-related factors (CABG, valve replacement, CPB duration) yielded inconclusive results due to extreme inter-study heterogeneity (*I*^2^ = 88–95%).

**Conclusion:**

Sepsis/infection, disease severity, advanced age, and elevated BMI are independent risk factors for DD in mechanically ventilated patients, whereas adequate nutritional status and preserved muscle strength are protective. Early identification of high-risk patients, nutritional optimisation, and infection control may reduce DD incidence.

**Systematic review registration:**

The publicly accessible registration URL is: https://www.crd.york.ac.uk/PROSPERO/view/CRD420261302793, this systematic review was registered with PROSPERO under the unique identifier CRD420261302793.

## 1 Introduction

Mechanical ventilation (MV) is a cornerstone of supportive care in the intensive care unit (ICU), yet it frequently induces rapid diaphragmatic atrophy and contractile dysfunction, collectively termed ventilator-induced diaphragm dysfunction (VIDD) (1). The principal features include diaphragmatic atrophy, sarcomeric injury, decreased contractile force, impaired endurance, and reduced excursion (2). VIDD is one of the major contributors to weaning failure (3). The reported incidence of diaphragmatic dysfunction (DD) in mechanically ventilated patients ranges from 60 to 80% (4), rising to as high as 80% in critically ill patients with prolonged intubation (5). DD progresses rapidly: diaphragm thickness decreases by approximately 9, 20, and 26% within 24, 48, and 72 h of MV, respectively (6). Its consequences include impaired respiratory function, ventilator dependence, ventilator-associated pneumonia (VAP), weaning failure, prolonged hospitalisation, and increased healthcare costs (7).

The pathophysiology of VIDD involves diaphragmatic disuse, oxidative stress, and activation of proteolytic pathways (8, 9). Despite this mechanistic understanding, the clinical risk factors for DD have not been systematically characterised. Available evidence implicates MV duration, ventilation mode, sedation depth, and sepsis (10), but the underlying studies are predominantly single-centre with small samples and inconsistent findings. A comprehensive, quantitative synthesis is lacking. The present systematic review and meta-analysis was therefore conducted to identify and quantify risk factors for DD in mechanically ventilated patients, with the aim of informing clinical risk stratification and preventive strategies. The study was registered on PROSPERO (CRD420261302793).

## 2 Materials and methods

### 2.1 Inclusion and exclusion criteria

#### 2.1.1 Study design

Case–control studies, cohort studies, and cross-sectional studies were eligible for inclusion.

#### 2.1.2 Participants

Adult mechanically ventilated patients (aged ≥ 18 years) who received MV for ≥ 24 h were included. No restrictions were applied to underlying disease type, disease severity, or ventilation mode.

#### 2.1.3 Exposure

Any potential risk factor associated with DD was considered.

#### 2.1.4 Outcome

The occurrence of DD, assessed using bedside ultrasonography.

#### 2.1.5 Exclusion criteria

Studies were excluded if they met any of the following criteria: ① duplicate publications; ② conference abstracts, reviews, or case reports; ③ animal experimental studies; ④ studies from which complete data could not be extracted; ⑤ studies with excessively low quality scores; or ⑥ publications in languages other than Chinese or English.

### 2.2 Search strategy

Two investigators independently searched seven electronic databases: PubMed, Embase, Web of Science, China National Knowledge Infrastructure (CNKI), Wanfang Database, VIP Database for Chinese Technical Periodicals, and the Chinese Biomedical Literature Database (CBM), from inception through July 2025. Reference lists of included studies were hand-searched for additional records. Search terms combined MeSH headings with free-text keywords covering “mechanical ventilation,” “diaphragm dysfunction/atrophy/weakness,” “ventilator-induced diaphragm dysfunction/VIDD,” and “risk factors/predictors/influencing factors.”

### 2.3 Study selection and data extraction

Two reviewers independently screened records and extracted data; discrepancies were resolved by a third reviewer. Screening proceeded in two stages: title and abstract screening after deduplication in Note Express, followed by full-text review against the prespecified criteria. Extracted items included: study characteristics (design, country, year of publication); participant demographics (sample size, age, sex, underlying diseases); DD diagnostic criteria and assessment method; effect estimates (ORs with 95% CIs, preferentially from multivariable analyses); and elements required for risk-of-bias scoring.

### 2.4 Risk of bias assessment

Risk of bias was assessed independently by two reviewers. Cohort and case–control studies were evaluated with the Newcastle-Ottawa Scale (NOS; maximum 9 points): scores ≥ 7 indicated low risk, 5–6 moderate risk, and ≤ 4 high risk. Cross-sectional studies were evaluated using the AHRQ checklist (maximum 11 points): scores 8–11 indicated high quality, 4–7 moderate quality, and 0–3 low quality. Only studies of moderate quality or above were retained.

### 2.5 Statistical analysis

Meta-analyses were performed using Review Manager 5.4. Multivariable-adjusted ORs with 95% CIs were used as the primary effect measure. Heterogeneity was assessed with the chi-square test and quantified by *I*^2^. A fixed-effects model was applied when *p* ≥ 0.10 and *I*^2^ ≤ 50%; a random-effects model was used when *p* < 0.10 or *I*^2^ > 50%. Statistical significance was set at *p* < 0.05. Risk factors reported by a single study were described narratively.

### 2.6 Protocol deviations

One deviation from the pre-registered PROSPERO protocol is disclosed. The registered protocol planned to pool all MV-related exposures in a single meta-analytic estimate. Following data extraction, this was judged methodologically unjustifiable given three mutually incompatible operationalizations; disaggregated narrative subgroups were therefore substituted.

## 3 Results

### 3.1 Literature screening process and results

**Figure 1:**
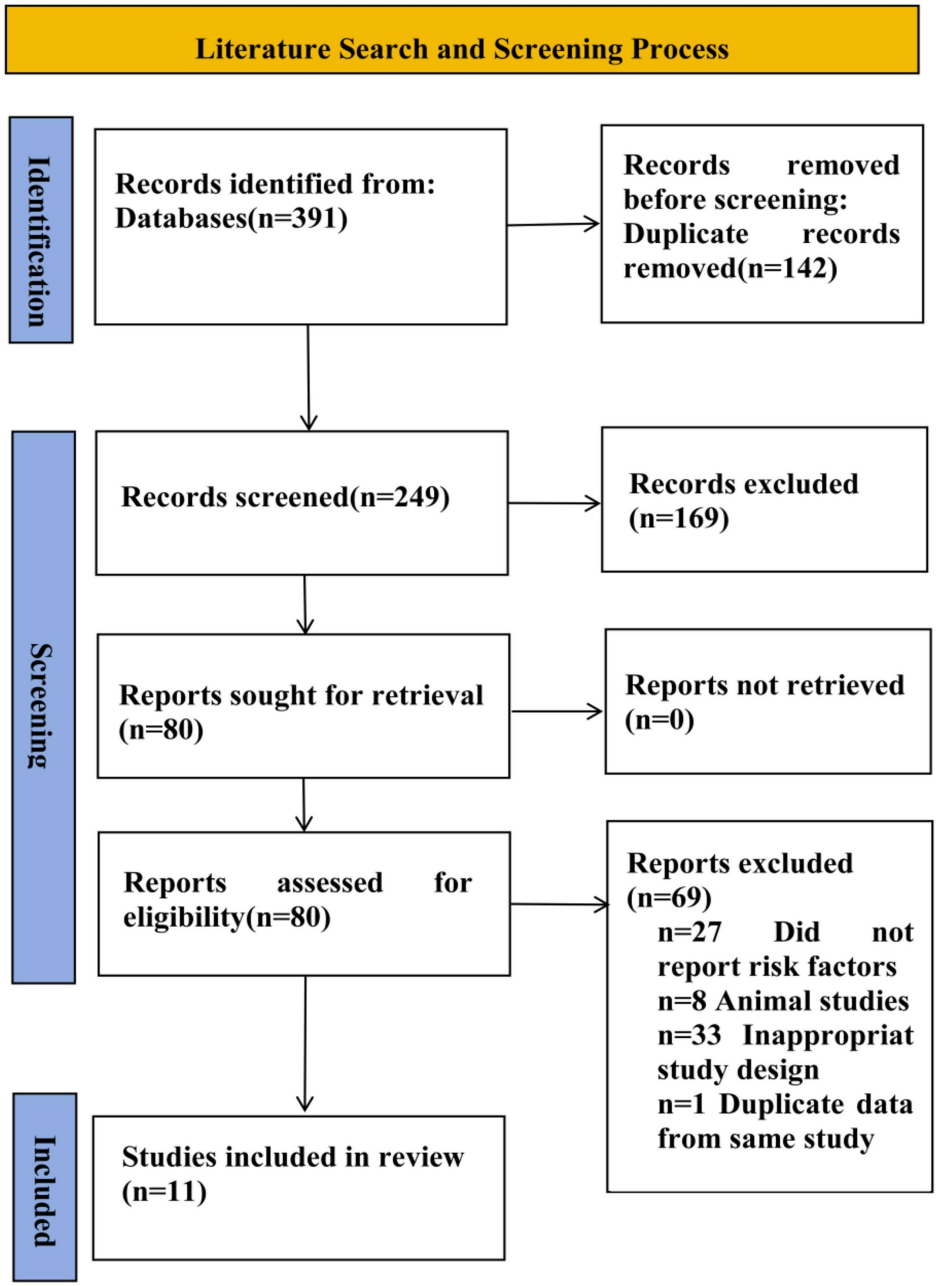
PRISMA flow diagram of the literature screening process.

The initial search identified 391 records. After deduplication, 249 remained. Title and abstract screening retained 80 articles for full-text review, of which 69 were excluded: 33 for ineligible study design, 8 were animal studies, 27 did not report DD risk factors, and 1 was a duplicate dataset. Eleven studies were included. The screening process is presented in Figure 1.

### 3.2 Characteristics of included studies

The 11 included studies (11–21) enrolled a cumulative sample of 5,272 mechanically ventilated patients. All studies assessed diaphragmatic function by bedside ultrasonography. Study populations comprised patients with severe pneumonia (*n* = 2), sepsis (*n* = 1), post-cardiac surgery (*n* = 4), and mixed ICU populations (*n* = 4). Reported risk factors spanned demographic, disease-severity, MV-related, surgical, pharmacological, and nutritional domains. Analytical approaches included univariable analysis, multivariable logistic regression, ROC analysis, and nomogram construction. Study characteristics are summarised in Table 1.

**Table 1:** Characteristics of the included studies.

Included study	Region	Study design	Sample size	Study population	DD diagnostic criteria	Diaphragmatic function assessment method	DD incidence (%)	Timing of DD assessment	Risk factors	Adjusted confounders
Bruni et al. (11)	Italy	Cohort study	100	Adult patients undergoing cardiac surgery	DTF < 20%	Color Doppler ultrasonography	38%	At first spontaneous breathing trial (SBT)	②③⑤⑥⑨⑩	Age, sex, diabetes, thyroid disease, type of surgery
Laghlam et al. (12)	France	Cohort study	3,577	Adult patients undergoing cardiac surgery	Diaphragm excursion: quiet breathing < 9 mm (female)/< 10 mm (male); sniff test < 16 mm (female)/< 18 mm (male); deep breathing < 37 mm (female)/< 47 mm (male)	Color Doppler ultrasonography	7.60%	Post-extubation	②⑤⑥⑨⑩	Age, sex, hypertension, diabetes, COPD, peripheral arterial disease, CPB duration, aorticcross-clamp time, etc.
Le Neindre (13)	France	Cohort study	50	Adult patients with sepsis	DTF < 20%	Color Doppler ultrasonography	58%	Within 24 h of sepsis/septic shock diagnosis; daily thereafter until ICU discharge or death	⑦⑧	—
Pu et al. (14)	China	Cohort study	126	Adult patients with expected MV ≥ 48 h	DTF < 20%	Color Doppler ultrasound diagnostic device	40.50%	DD assessed daily by ultrasound during MV	①⑫⑬	Age, sex, prior nutritional status, caloric intake, amino acid intake, acute disease severity, MV mode
Bai (15)	China	Cohort study	221	Adult ICU patients	DTF < 20%	Color Doppler ultrasound diagnostic device	57%	Day 1, 3, and 5 after ICU admission	⑦⑧⑯⑮	Serum calcium concentration, CRRT, prone positioning, shock, BMI, smoking history, alcohol history, blood urea nitrogen, serum calcium, enteral nutrition, vasoactive agents, insulin
Li et al. (16)	China	Cross-sectional study	150	Adult ICU patients on MV within 48 h	DTF < 20%, or diaphragm excursion < 1 cm, or diaphragm thickness < 0.2 cm	Color Doppler ultrasound diagnostic device	49.33%	Within 48 h of MV initiation	④⑪⑱	Age, sex, BMI, APACHE II score, diabetes, hypertension, white blood cell count, neutrophil count
Tao (17)	China	Cross-sectional study	304	Adult patients after cardiac surgery	Diaphragm excursion < 10 mm	Color Doppler ultrasound diagnostic device	31.25%	DD assessed at SBT discharge	②⑧③⑰⑲	Platelet count, APACHE II score
Wang et al. (18)	China	Cohort study	261	Adult patients with severe pneumonia on MV; endotracheal intubation MV ≥ 24 h	DTF < 20%, or diaphragm excursion < 1 cm, or diaphragm thickness < 0.2 cm	Color Doppler ultrasound diagnostic device	43.96%	Within 24 h of MV initiation	①④⑦	Early functional exercise, MV duration, sex, PaO₂
Wu et al. (19)	China	Cross-sectional study	70	Adult ICU patients on MV ≥ 72 h	Diaphragm excursion < 10 mm	Color Doppler ultrasound diagnostic device	60%	Within 72 h of MV initiation	①②⑧⑨⑪	Sex, MV mode, age, grip strength
Zhao et al. (20)	China	Case–control study	132	Adult patients with severe pneumonia on MV	DTF < 20%	Color Doppler ultrasound diagnostic device	60.61%	Day 1–4 of invasive MV	①⑧⑨⑭	Age, intra-abdominal pressure
Yang et al. (21)	China	Cohort study	281	Adult patients after cardiac surgery with CPB	DTF < 20%, or diaphragm excursion < 1 cm	Color Doppler ultrasound diagnostic device	11.40%	Daily during postoperative ICU stay	②㉑㉒	Age, hypertension, cardiac insufficiency, type of surgery, CPB duration

Risk factor codes: ① APACHE II score; ② BMI: body mass index; ③ CPB duration; ④ serum albumin; ⑤ cardiac valve replacement surgery; ⑥ coronary artery bypass grafting; ⑦ infection/sepsis; ⑧ mechanical ventilation; ⑨ age; ⑩ diabetes mellitus; ⑪ grip strength; ⑫ SOFA score; ⑬ amino acid intake; ⑭ number of combined sedative agents; ⑮ Glasgow Coma Scale score; ⑯ insulin use; ⑰ dexmedetomidine dosage; ⑱ nutritional risk score; ⑲ pulmonary arterial hypertension; ㉑ left ventricular ejection fraction; ㉒ postoperative blood glucose.

### 3.3 Risk of bias assessment results

All seven cohort studies received NOS scores > 6, indicating low risk of bias. The case–control study was similarly rated as low risk. The three cross-sectional studies scored 7–8 on the AHRQ checklist, indicating moderate methodological quality. Detailed scores are presented in Tables 2–4.

**Table 2:** Risk of bias assessment results for the included case–control study (scores).

Included study	Selection of cases and controls	Comparability	Exposure	Total score
	Adequate definition and diagnosis of cases	Representativeness of cases	Selection of controls	Definition of controls
Zhao et al. (20)	1	1	1	1

**Table 3:** Risk of bias assessment results for the included cohort studies (scores).

Included study	Selection	Comparability	Outcome	Total score
	Representativeness of exposed cohort	Selection of non-exposed cohort	Ascertainment of exposure	Demonstration that outcome was not present at start
Bai (15)	1	1	1	0
Wang (18)	1	1	1	1
Aymeric et al. (13)	1	1	1	0
Hong et al. (14)	1	1	1	1
Driss et al. (12)	1	1	1	1
Andrea et al. (11)	1	1	1	1
Yang et al. (21)	1	1	1	0

**Table 4:** Risk of bias assessment results for the included cross-sectional studies.

Included study	①	②	③	④	⑤	⑥	⑦	⑧	⑨	⑩	⑪	Total score
Wu et al. (19)	Yes	Yes	Yes	Yes	No	Yes	No	Yes	No	Yes	No	7
Li et al. (16)	Yes	Yes	Yes	Yes	No	Yes	Yes	Yes	No	Yes	No	8
Tao (17)	Yes	Yes	Yes	Yes	No	Yes	Yes	Yes	No	Yes	No	8

① Whether the data source was clearly defined; ② Whether inclusion and exclusion criteria for the exposed and non-exposed groups (cases and controls) were listed or referenced from previous publications; ③ Whether the time period for participant recruitment was clearly stated; ④ Whether the study participants were representative; ⑤ Whether it was clarified that the measured variables were not masked by other characteristics; ⑥ Whether any quality assurance assessments were described; ⑦ Whether excluded participants were described in the outcome analysis; ⑧ Whether measures to assess and/or control for confounders were described; ⑨ Whether handling of missing data was explained; ⑩ Whether patient response rates and completeness of data collection were summarized; ⑪ If follow-up was conducted, whether the percentage of incomplete data or follow-up outcomes was reported.

### 3.4 Meta-analysis results

Twenty-two risk factors were reported across the 11 included studies. Based on clinical domain, they were categorised as: demographic characteristics, disease severity, MV-related factors, surgery-related factors, pharmacological treatment, nutritional status, and underlying disease. Factors reported by at least two studies were meta-analysed; single-study findings were described narratively.

#### 3.4.1 Demographic characteristics

Age and body mass index (BMI) were each reported as demographic risk factors for DD. For age, four studies were included (11, 12, 19, 20) (*I*^2^ = 0%, *p* = 0.65). Increasing age was a significant risk factor for DD (OR = 1.02, 95% CI: 1.00–1.04, *p* = 0.02). For BMI, five studies reported this factor (11, 12, 17, 19, 21); four provided continuous-variable data that were pooled (*I*^2^ = 0%, *p* = 0.47), showing that higher BMI was a significant risk factor (OR = 1.03, 95% CI: 1.01–1.06, *p* = 0.02). One additional study (17) analysed BMI ≥ 28 kg/m^2^ as a dichotomous variable and confirmed obesity as an independent risk factor for DD in post-cardiac surgery patients (OR = 3.70, 95% CI: 1.38–9.93, *p* = 0.009).

#### 3.4.2 Disease severity scores

Three disease severity instruments were examined: APACHE II, SOFA, and Glasgow Coma Scale (GCS). Higher APACHE II scores were a significant risk factor for DD (four studies (14, 18–20); *I*^2^ = 0%, *p* = 0.95; OR = 1.19, 95% CI: 1.09–1.30, *p* < 0.0001). The SOFA score was also independently associated with DD (OR = 1.79, 95% CI: 1.16–2.75, *p* = 0.008) (14), with an effect size larger than that of APACHE II, possibly reflecting the SOFA score’s ability to capture dynamic progression of sepsis-related organ damage (22). GCS was examined in one study (20): lower GCS scores were an independent risk factor for DD (OR = 0.79, 95% CI: 0.67–0.93, *p* = 0.004), with the direction consistent with GCS as a protective factor at higher values.

#### 3.4.3 Mechanical ventilation-related factors

Five studies examined MV-related exposures as risk factors for DD using four distinct operationalizations; the high definitional heterogeneity precluded pooled quantitative analysis. Regarding MV presence versus absence, Bai et al. (15) identified a non-significant trend toward increased DD risk among MV patients in a mixed ICU population (OR = 2.903, 95% CI: 0.801–11.37; *p* = 0.111). Le Neindre et al. (13) reported a significantly higher DD incidence in mechanically ventilated sepsis patients compared with non-ventilated patients (71% vs. 37%, *p* = 0.04); however, as no multivariable-adjusted OR was reported, formal pooling with the estimate from Bai et al. (15) was not performed. Regarding MV duration, Tao (17) identified prolonged MV as an independent predictor of DD in post-cardiac surgery patients (OR = 3.258, 95% CI: 1.801–5.894; *p* < 0.001). Regarding ventilation mode, Wu et al. (19) reported a lower DD risk in the invasive MV group compared with the non-invasive MV group (OR = 0.032, 95% CI: 0.002–0.507; *p* = 0.015); however, as patients receiving non-invasive MV generally present with milder illness and better-preserved airway protective reflexes, a lower DD incidence would theoretically be expected in this group. The observed inverse association may therefore reflect baseline differences in disease severity between groups rather than a true protective effect of invasive ventilation. Zhao et al. (20) observed no significant difference in DD risk between A/C and SIMV/PSV modes among patients with severe pneumonia (OR = 1.439, 95% CI: 0.614–3.373; *p* = 0.403) (Table 5). It should be noted that the included studies are subject to varying degrees of reverse causation bias or residual confounding by disease severity.

**Table 5:** Meta-analysis results of risk factors for DD in mechanically ventilated patients.

Outcome measure	Unit per OR	No. of studies	Heterogeneity test		Effect model	Meta-analysis result	*p*
	Unit per OR		*p*	*I* ^2^		OR (95% CI)	
Age (11, 12, 19, 20)	Per 1 year	4	0.65	0%	Fixed	1.02 (1.00, 1.04)	0.02
BMI (11, 12, 19, 21)	Per 1 kg/m^2^	4	0.47	0%	Fixed	1.03 (1.01, 1.06)	0.02
APACHE II score (14, 18–20)	Per 1 point	4	0.95	0%	Fixed	1.19 (1.09, 1.30)	< 0.0001
CABG (11, 12)	—	2	< 0.001	95%	Random	0.63 (0.07, 6.04)	0.69
Valve replacement surgery (11, 12)	—	2	0.006	92%	Random	1.12 (0.82, 1.51)	0.48
CPB duration (11, 17)	Per 1 min	2	0.003	88%	Random	1.02 (0.98, 1.07)	0.26
Serum albumin (16, 18)	Per 1 g/L	2	0.08	67%	Random	0.88 (0.79, 0.98)	0.018
Grip strength (16, 19)	Per 1 kg	2	0.16	48%	Fixed	0.70 (0.62, 0.78)	< 0.0001
Diabetes mellitus (11, 12)	—	2	0.86	0%	Fixed	0.88 (0.70, 1.11)	0.3
Infection/sepsis (13, 15, 18)	—	3	0.93	0%	Fixed	2.65 (1.32, 5.34)	0.006

OR, odds ratio; 95% CI, 95% confidence interval; *I*^2^, heterogeneity statistic; APACHE II, Acute physiology and chronic health evaluation II; BMI, body mass index; CABG, Coronary artery bypass grafting; CPB, Cardiopulmonary bypass.

#### 3.4.4 Surgery-related factors

Surgery-related factors examined included CABG (11, 12), valve replacement surgery (11, 12), and CPB duration (11, 17). Each was reported by two studies; however, inter-study heterogeneity was extreme (*I*^2^ = 95, 92, and 88%, respectively) and none of the pooled estimates reached statistical significance (all *p* > 0.05). The primary driver of heterogeneity was the discrepancy in DD screening strategy and diagnostic criterion between Bruni et al. (11) and Laghlam et al. (12): the former screened systematically at the first spontaneous breathing trial using DTF < 20% and reported 38% DD incidence, whereas the latter screened symptomatically after extubation using reduced diaphragmatic displacement and reported 7.6% incidence. Given this level of heterogeneity, pooled estimates for surgery-related factors should not be interpreted as stable effect sizes.

#### 3.4.5 Pharmacological treatment factors

Among pharmacological factors, sedative agents, opioids, corticosteroids, and insulin were reported. Three studies examined sedative agents (13, 17, 20); heterogeneous exposure definitions precluded pooling. Le Neindre et al. (13) found sedative use to be a risk factor for DD in sepsis patients (OR = 4.55, 95% CI: 1.23–16.88, *p* = 0.04). In contrast, Zhao et al. (20) and Tao (17) reported protective associations for combined sedative agents (OR = 0.76, 95% CI: 0.19–0.91, *p* = 0.012) and intraoperative dexmedetomidine dosage (OR = 0.997, 95% CI: 0.995–0.999, *p* < 0.001), respectively, suggesting agent-specific and context-specific effects. Tao (17) additionally reported corticosteroids as a risk factor (OR = 6.67, 95% CI: 1.61–27.66, *p* = 0.009) and intraoperative insulin use as a protective factor (OR = 0.07, 95% CI: 0.01–0.44, *p* = 0.005). These findings are described narratively and summarised in Table 5.

#### 3.4.6 Nutritional status

Among nutritional status-related factors, nutritional risk, serum albumin, amino acid intake, and grip strength were reported to be associated with DD, with all results suggesting that adequate nutritional status is an important protective factor. Regarding serum albumin, two studies were included (16, 18), with moderate heterogeneity (*I*^2^ = 67%, *p* = 0.08). The meta-analysis demonstrated that higher serum albumin levels were a protective factor against DD in mechanically ventilated patients (OR = 0.88, 95% CI: 0.84–0.94, *p* = 0.018). Regarding grip strength, two studies were included (16, 19), with moderate heterogeneity (*I*^2^ = 48%, *p* = 0.16). The pooled results similarly showed that higher grip strength was a protective factor (OR = 0.70, 95% CI: 0.62–0.78, *p* < 0.0001). Additionally, Li et al. (16) found that the presence of nutritional risk increased the risk of DD (OR = 5.76, 95% CI: 2.20–15.10, *p* < 0.001), and Pu et al. (14) found that early adequate amino acid intake was a protective factor (OR = 0.49, 95% CI: 0.32–0.76, *p* = 0.001).

#### 3.4.7 Underlying disease-related factors

Among underlying disease factors, infection/sepsis was the most consistently reported risk factor: three studies (13, 15, 18) yielded a significant pooled estimate with low heterogeneity (*I*^2^ = 0%, *p* = 0.93; OR = 2.65, 95% CI: 1.32–5.34, *p* = 0.006). Diabetes mellitus was reported by two post-cardiac surgery studies (11, 12) (*I*^2^ = 0%, *p* = 0.86) but showed no significant association (OR = 0.88, 95% CI: 0.70–1.11, *p* = 0.30). Postoperative hyperglycaemia following CPB was an independent risk factor in one study (21) (OR = 3.27, 95% CI: 2.58–5.32, *p* < 0.001). Pulmonary arterial hypertension (11, 12) and hypertension (11, 12) were each reported by two studies but with high heterogeneity, precluding reliable pooled estimates. Full narrative details are presented in Table 5.

## 4 Discussion

This review synthesises evidence on DD risk factors across six clinical domains. The most robustly supported findings converge on a picture of DD as a multifactorial syndrome in which systemic illness severity, inflammatory injury, and nutritional depletion interact to accelerate diaphragmatic wasting. Infection/sepsis, disease severity scores (APACHE II, SOFA), advanced age, and elevated BMI each independently increase risk, while adequate nutritional status and preserved muscle strength are protective. Surgery-related and MV-related factors showed clinically plausible but methodologically unresolvable signals. Pharmacological exposures exhibit agent-specific directionality.

### 4.1 Disease severity and diaphragmatic dysfunction

Disease severity scores were among the most consistent and robustly poolable predictors of DD in this review. The APACHE II score integrates acute physiological parameters, age, and chronic health status; higher values signal worsening compensatory capacity and a catabolic milieu that promotes diaphragmatic protein degradation (23). The SOFA score captures dynamic organ failure progression and carries a larger effect size than APACHE II in the available data, consistent with its sensitivity to evolving sepsis-related dysfunction (22). GCS, as a measure of neurological status, likely reflects both sedation depth and the severity of the underlying neurological injury, each of which may independently impair respiratory drive and diaphragmatic activation (24). Collectively, disease severity scoring provides a clinically accessible substrate for early DD risk stratification: patients presenting with high APACHE II or SOFA scores at ICU admission should be considered high-priority candidates for prospective diaphragmatic monitoring.

### 4.2 Mechanical ventilation and diaphragmatic dysfunction

MV is mechanistically linked to DD through three pathways: diaphragmatic disuse atrophy, proteolytic activation via the ubiquitin-proteasome, calpain, and autophagy-lysosome systems, and oxidative stress driven by mitochondrial calcium overload and diaphragmatic hypoperfusion (25–27). This mechanistic framework predicts that ventilation mode, duration, and intensity each independently modulate DD risk; however, this prediction could not be empirically confirmed in the present review, as the five included studies operationalised MV exposure in mutually incompatible ways. The inability to pool MV-related exposures reflects not merely a limitation of this review but a structural gap in the observational literature. Existing studies variously define MV exposure as a binary variable, a duration threshold, or a mode category, each capturing a fundamentally different clinical dimension. As Dres et al. have noted, the absence of consensus definitions for MV-related exposures has persistently impeded cumulative evidence synthesis in DD research (28). Despite the lack of poolable effect estimates, the directional pattern across studies is consistent with mechanistic expectations. The non-significant trend toward increased DD risk with MV presence and the significant association with prolonged MV duration are both compatible with a dose-dependent disuse mechanism, even though confounding and reverse causation preclude causal interpretation. From a preventive standpoint, minimising the duration of fully controlled ventilation, transitioning to assisted modes that preserve spontaneous respiratory effort as early as clinically feasible, and incorporating early spontaneous breathing trials constitute a diaphragm-protective ventilation strategy supported by both mechanistic evidence and expert consensus (29). Future observational studies should pre-specify harmonised MV exposure definitions, examine duration, mode, and intensity as separate predictors, and adopt designs in which exposure measurement precedes DD assessment.

### 4.3 Nutritional status and diaphragmatic dysfunction

The protective association between nutritional status and DD is biologically coherent. Serum albumin reflects not only protein synthetic capacity but also antioxidant defence, colloid osmotic pressure maintenance, and nutrient transport (30); hypoalbuminaemia signals heightened catabolism and systemic inflammation, both of which accelerate diaphragmatic wasting (31). Adequate amino acid intake supports *de novo* synthesis of contractile proteins at a time when proteolytic drive is highest. Grip strength, measurable at the bedside within minutes, integrates global skeletal muscle reserve and may serve as an early, actionable warning signal for DD risk. Translating these findings into practice, current ESPEN guidelines for critically ill patients recommend initiating enteral nutrition within 24–48 h of ICU admission, titrating protein delivery to ≥ 1.3 g/kg/day by day 3–5, and incorporating regular assessment of functional muscle endpoints (grip strength, ultrasound-measured diaphragm thickness fraction) to guide nutritional adequacy. The present data support prioritising these measures specifically in patients identified as high-risk for DD at admission.

### 4.4 Surgery-related factors and diaphragmatic dysfunction

The meta-analysis of surgery-related factors was unable to delineate the independent effects of specific surgical types due to high inter-study heterogeneity. The heterogeneity primarily originated from methodological differences between studies: Bruni et al. (11) employed a systematic screening strategy, diagnosing DD at the time of the first spontaneous breathing trial using a thickening fraction < 20% as the criterion, and reported an incidence of 38%, identifying CPB duration as the primary risk factor. In contrast, Laghlam et al. (12) adopted a symptom-based screening approach, diagnosing DD after extubation based on reduced diaphragmatic displacement, and reported an incidence of 7.6%, identifying coronary artery bypass grafting as the primary risk factor. The two studies exhibited notable discrepancies in diagnostic strategies, timing of assessment, diagnostic criteria, and conclusions regarding risk factors. Existing evidence suggests that cardiac surgery can lead to diaphragmatic injury through multiple mechanisms: intraoperative thoracotomy physically restricts diaphragmatic movement; the phrenic nerve may sustain direct or indirect injury during surgical manipulation; and the systemic inflammatory response triggered by the initiation of CPB can affect the respiratory muscles (32). Therefore, post-cardiac surgery patients represent a high-priority target for prospective diaphragmatic monitoring. Protocol-driven bedside ultrasound assessment of diaphragm at the first spontaneous breathing trial would allow early identification of post-surgical DD and timely initiation of inspiratory muscle training.

### 4.5 Pharmacological factors and diaphragmatic dysfunction

The pharmacological findings highlight the importance of agent-specific rather than class-level reasoning about sedation and DD. Conventional sedative agents such as propofol suppress respiratory drive and attenuate spontaneous diaphragmatic activity, contributing to disuse atrophy (33); opioids act similarly via central respiratory depression (34). Dexmedetomidine, by contrast, is a selective *α*₂-adrenergic agonist that preserves spontaneous breathing, and its association with lower DD risk in the available data (17) is consistent with a diaphragm-sparing mechanism (35). Corticosteroids showed a risk-increasing association, likely reflecting steroid myopathy superimposed on critical illness myopathy. Intraoperative insulin was protective, possibly by attenuating hyperglycaemia-induced oxidative damage to contractile proteins. Clinically, these findings support sedation protocols that minimise conventional sedative exposure, preferentially incorporate agents with respiratory-sparing properties, avoid non-essential corticosteroids, and maintain perioperative glycaemic control as components of a DD-prevention bundle.

### 4.6 Infection/sepsis and diaphragmatic dysfunction

Infection and sepsis emerged as the most consistently poolable non-MV risk factor for DD, with a stable pooled estimate across three studies of different designs and settings (OR = 2.65; *I*^2^ = 0%). The mechanistic basis is well characterised: pro-inflammatory cytokines impair contractile protein function and disrupt neuromuscular transmission; simultaneous activation of the ubiquitin-proteasome, calpain, and caspase systems drives rapid myofibrillar protein degradation; oxidative stress from excessive reactive oxygen species perpetuates contractile protein damage; mitochondrial dysfunction reduces ATP availability; and microcirculatory failure limits oxygen and nutrient delivery (36, 37). The clinical implication is direct: timely source control, targeted antimicrobial therapy, and adjunctive anti-inflammatory and antioxidant strategies are not only treatments for sepsis but also potentially disease-modifying interventions for the prevention of DD.

This review establishes that DD in mechanically ventilated patients is driven by converging pathways of systemic illness severity, inflammatory injury, and nutritional depletion, and is attenuated by preserved nutritional and muscular reserve. The practical implication is a multicomponent prevention strategy implementable within existing ICU workflows: systematic DD risk stratification at admission using APACHE II and SOFA scores; early enteral nutrition with adequate protein delivery (≥ 1.3 g/kg/day) guided by serial grip strength and albumin monitoring; aggressive source control and timely antimicrobial therapy in patients with infection or sepsis; and sedation protocols that minimise conventional sedative exposure and incorporate respiratory-sparing agents where feasible. Post-cardiac surgery patients warrant particular attention given the high incidence reported in this population and the potential modifiability of CPB duration and perioperative glycaemic control.

The field now requires prospective studies that pre-specify harmonised DD diagnostic criteria, standardise exposure assessment timing relative to intubation, and power subgroup analyses by clinical setting and ventilation mode. Interventional trials testing nutrition-optimisation and sedation-minimisation bundles against DD incidence as a primary endpoint would provide the causal evidence that observational synthesis cannot.

Several limitations should be considered when interpreting these findings. First, most risk factors were reported by only two studies, constraining the statistical precision of pooled estimates and the feasibility of comprehensive subgroup analyses. Second, diagnostic criteria and assessment timing for DD differed across studies: although all used bedside ultrasonography, specific thresholds (DTF < 20% versus excursion < 1 cm versus composite criteria) and assessment time points varied, limiting cross-study comparability. Third, MV-related exposures were operationalised in three different ways across five studies, preventing pooling and limiting conclusions about specific MV-related risk factors; future research should pre-specify a harmonised operational definition. Future high-quality prospective studies with pre-specified exposure definitions, standardised DD diagnostic criteria, and assessment timing relative to exposure measurement are needed to establish causal relationships and to evaluate targeted preventive strategies.

## Data Availability

The original contributions presented in the study are included in the article/supplementary material, further inquiries can be directed to the corresponding author.

## References

[1] Powers SK. Ventilator-induced diaphragm dysfunction: phenomenology and mechanism(s) of pathogenesis. J Physiol. (2024) 602:4729–52. doi: 10.1113/JP283860, 39216087

[2] Sheng Y Wang T Zhang X Shao W Wang Y Kang X . Effects of external diaphragmatic pacing with neurally adjusted ventilatory assist on diaphragm function in AECOPD patients. Sci Rep. (2025) 15:19340. doi: 10.1038/s41598-025-04352-2, 40456822 PMC12130502

[3] Saleem Khan K Meaney J Martin-Loeches I Collins DV. MRI assessment of global and regional diaphragmatic motion in critically ill patients following prolonged ventilator weaning. Med Sci (Basel). (2019) 7:66. doi: 10.3390/medsci7050066, 31121866 PMC6571928

[4] Supinski GS Morris PE Dhar S Callahan LA. Diaphragm dysfunction in critical illness. Chest. (2018) 153:1040–51. doi: 10.1016/j.chest.2017.08.1157, 28887062 PMC6026291

[5] Vetrugno L Guadagnin GM Barbariol F Langiano N Zangrillo A Bove T. Ultrasound imaging for diaphragm dysfunction: a narrative literature review. J Cardiothorac Vasc Anesth. (2019) 33:2525–36. doi: 10.1053/j.jvca.2019.01.003, 30686657

[6] Schepens T Verbrugghe W Dams K Corthouts B Parizel PM Jorens PG. The course of diaphragm atrophy in ventilated patients assessed with ultrasound: a longitudinal cohort study. Crit Care. (2015) 19:422. doi: 10.1186/s13054-015-1141-0, 26639081 PMC4671211

[7] Dres M Goligher EC Heunks LMA Brochard LJ. Critical illness-associated diaphragm weakness. Intensive Care Med. (2017) 43:1441–52. doi: 10.1007/s00134-017-4928-4, 28917004

[8] Liu Y Li L. Ventilator-induced diaphragmatic dysfunction in critical illness. Exp Biol Med (Maywood). (2018) 243:1329–37. doi: 10.1177/1535370218811950, 30453774 PMC6348597

[9] Fu W Guan L Liu Q Xie Z You J Chen R. Ventilator-induced diaphragmatic dysfunction: pathophysiology, monitoring and advances in potential treatment and prevention. Eur Respir Rev. (2025) 34:250069. doi: 10.1183/16000617.0069-2025, 41062169 PMC12505153

[10] Zambon M Beccaria P Matsuno J Gemma M Frati E Colombo S . Mechanical ventilation and diaphragmatic atrophy in critically ill patients: an ultrasound study. Crit Care Med. (2016) 44:1347–52. doi: 10.1097/CCM.0000000000001657, 26992064

[11] Bruni A Garofalo E Pasin L Serraino GF Cammarota G Longhini F . Diaphragmatic dysfunction after elective cardiac surgery: a prospective observational study. J Cardiothorac Vasc Anesth. (2020) 34:3336–44. doi: 10.1053/j.jvca.2020.06.038, 32653270

[12] Laghlam D Lê MP Srour A Monsonego R Estagnasié P Brusset A . Diaphragm dysfunction after cardiac surgery: reappraisal. J Cardiothorac Vasc Anesth. (2021) 35:3241–7. doi: 10.1053/j.jvca.2021.02.023, 33736912

[13] Le Neindre A Wormser J Luperto M Bruel C Misset B Bouhemad B . Diaphragm function in patients with sepsis and septic shock: a longitudinal ultrasound study. Aust Crit Care. (2023) 36:239–46. doi: 10.1016/j.aucc.2022.01.003, 35272911

[14] Pu H Doig GS Lv Y Wu X Yang F Zhang S . Modifiable risk factors for ventilator associated diaphragmatic dysfunction: a multicenter observational study. BMC Pulm Med. (2023) 23:343. doi: 10.1186/s12890-023-02633-y, 37700263 PMC10498609

[15] Bai YF. Construction of a Risk Prediction Model for Diaphragmatic Dysfunction in Critically ill Patients [Dissertation]. Beijing: Peking Union Medical College (2024).

[16] Li JL Lu ZH Liu N Zhang J Zhang R. Correlation analysis of nutritional risk, grip strength and diaphragmatic function in ICU mechanically ventilated patients. J Southeast Univ (Med Sci Ed). (2025) 44:860–5. doi: 10.3969/j.issn.1671-6264.2025.05.023

[17] Tao YY. Investigation of the Current Status of Diaphragmatic Dysfunction after Cardiac Surgery and Construction of a Prediction Model [Dissertation]. Fuzhou: Fujian Medical University (2023).

[18] Wang XC Huang QM Zhang H Qin HJ Ye H Zhu YX . Construction and validation of a risk prediction model for ventilator-induced diaphragmatic dysfunction within 24 hours in patients with severe pneumonia on mechanical ventilation. China Med Herald. (2024) 21. doi: 10.20047/j.issn1673-7210.2024.26.25

[19] Wu YH Yang R Xi F Liang HX Zhai ML Yang B. Correlation analysis of grip strength level and diaphragmatic function in ICU mechanically ventilated patients. Chin J Nurs Res. (2022) 36:345–8. doi: 10.12102/j.issn.1009-6493.2022.02.031

[20] Zhao XL Ning JJ Zhong J Wang HH Hou LM Li HB. Analysis of influencing factors of diaphragmatic dysfunction in mechanically ventilated patients with severe pneumonia. J Youjiang Med Univ Nationalities. (2025) 47:720–3. doi: 10.3969/j.issn.1001-5817.2025.04.030

[21] Yang XP Shi Y Pei FB Zhang SM Ma H Han ZQ . Risk factors for diaphragmatic insufficiency after cardiopulmonary bypass in cardiac surgery: a retrospective cohort study. Chin J Clin Thorac Cardiovasc Surg. (2025) 32:1140–5. doi: 10.7507/1007-4848.202410012

[22] Tekin B Kılıç J Taşkın G Solmaz I Tezel O Başgöz BB. The comparison of scoring systems: SOFA, APACHE-II, LODS, MODS, and SAPS-II in critically ill elderly sepsis patients. J Infect Dev Ctries. (2024) 18:122–30. doi: 10.3855/jidc.18526, 38377099

[23] Koçak A Urfalı S. A retrospective analysis of the predictive role of RDW, MPV, and MPV/PLT values in 28-day mortality of geriatric sepsis patients: associations with APACHE II and SAPS II scores. Medicina (Kaunas). (2025) 61:318. doi: 10.3390/medicina61081318, 40870363 PMC12388282

[24] Mehta R Chinthapalli K. Glasgow coma scale explained. BMJ. (2019) 365:l1296. doi: 10.1136/bmj.l1296, 31048343

[25] Picard M Jung B Liang FX Azuelos I Hussain S Goldberg P . Mitochondrial dysfunction and lipid accumulation in the human diaphragm during mechanical ventilation. Am J Respir Crit Care Med. (2012) 186:1140–9. doi: 10.1164/rccm.201206-0982OC, 23024021

[26] Zhao X Liu Y Wang D Li T Xu Z Li Z . Role of GLP-1 receptor agonists in sepsis and their therapeutic potential in sepsis-induced muscle atrophy (review). Int J Mol Med. (2025) 55:1–16. doi: 10.3892/ijmm.2025.5515, 40052580 PMC11936484

[27] Liu Z Yu Q Zhou X Ma H Wei R Xia J. Carnosic acid attenuates ventilator-induced diaphragmatic dysfunction via activation of the Nrf2/HO-1 pathway. Int Immunopharmacol. (2026) 168:115926. doi: 10.1016/j.intimp.2025.115926, 41275825

[28] Dres M Demoule A. Diaphragm dysfunction during weaning from mechanical ventilation: an underestimated phenomenon with clinical implications. Crit Care. (2018) 22:48. doi: 10.1186/s13054-018-1992-2, 29558983 PMC5861656

[29] Goligher EC Jonkman AH Dianti J Vaporidi K Beitler JR Patel BK . Clinical strategies for implementing lung and diaphragm-protective ventilation: avoiding insufficient and excessive effort. Intensive Care Med. (2020) 46:2314–26. doi: 10.1007/s00134-020-06288-9, 33140181 PMC7605467

[30] Neel DR McClave S Martindale R. Hypoalbuminaemia in the perioperative period: clinical significance and management options. Best Pract Res Clin Anaesthesiol. (2011) 25:395–400. doi: 10.1016/j.bpa.2011.07.006, 21925404

[31] Allison SP Lobo DN. The clinical significance of hypoalbuminaemia. Clin Nutr. (2024) 43:909–14. doi: 10.1016/j.clnu.2024.02.018, 38394971

[32] Boussuges M Blanc P Bregeon F Boussuges A. Interest of thoracic ultrasound after cardiac surgery or interventional cardiology. World J Cardiol. (2024) 16:118–25. doi: 10.4330/wjc.v16.i3.118, 38576518 PMC10989224

[33] Liu Q Li Q Qiao D Chen Y Zheng L Zhao C . The effect of remimazolam combined with low-dose propofol on diaphragmatic function in patients undergoing hysteroscopic surgery: a randomized clinical trial. BMC Anesthesiol. (2025) 25:625. doi: 10.1186/s12871-025-03524-x, 41275085 PMC12754893

[34] Qiao D Liu W Xue H Liu R Gao Y Dong J . Comparison of the effects of esketamine/midazolam and remifentanil/midazolam on respiratory mechanics in mechanically ventilated patients with acute respiratory distress syndrome. BMC Anesthesiol. (2025) 25:339. doi: 10.1186/s12871-025-03211-x, 40618022 PMC12229023

[35] Pearson SD Patel BK. Evolving targets for sedation during mechanical ventilation. Curr Opin Crit Care. (2020) 26:47–52. doi: 10.1097/MCC.0000000000000687, 31764193 PMC8086012

[36] Petrof BJ. Diaphragm weakness in the critically ill: basic mechanisms reveal therapeutic opportunities. Chest. (2018) 154:1395–403. doi: 10.1016/j.chest.2018.08.1028, 30144420

[37] Liu H Wu J Yao J Wang H Li ST. The role of oxidative stress in decreased acetylcholinesterase activity at the neuromuscular junction of the diaphragm during sepsis. Oxidative Med Cell Longev. (2017) 2017:9718615. doi: 10.1155/2017/9718615, 29230271 PMC5694580

